# Single-cell analysis reveals individual spore responses to simulated space vacuum

**DOI:** 10.1038/s41526-018-0059-7

**Published:** 2018-12-04

**Authors:** Lin He, Shiwei Wang, Marta Cortesão, Muying Wu, Ralf Moeller, Peter Setlow, Yong-qing Li

**Affiliations:** 1 School of Electrical Engineering and Intelligentization, Dongguan University of Technology, Dongguan, Guangdong China; 2 Department of Physics, East Carolina University, Greenville, North Carolina 27858-4353 USA; 3 School of Chemical Engineering and Energy Technology, Dongguan University of Technology, Dongguan, China; 4 Space Microbiology Research Group, Departmet of Radiation Biology, Institute of Aerospace Medicine, German Aerospace Center (DLR e.V.), Cologne (Koeln), Germany; 5 Department of Molecular Biology and Biophysics, UConn Health, Farmington, Connecticut 06030-3305 USA

## Abstract

Outer space is a challenging environment for all forms of life, and dormant spores of bacteria have been frequently used to study the survival of terrestrial life in a space journey. Previous work showed that outer space vacuum alone can kill bacterial spores. However, the responses and mechanisms of resistance of individual spores to space vacuum are unclear. Here, we examined spores’ molecular changes under simulated space vacuum (~10^−5^ Pa) using micro-Raman spectroscopy and found that this vacuum did not cause significant denaturation of spore protein. Then, live-cell microscopy was developed to investigate the temporal events during germination, outgrowth, and growth of individual *Bacillus* spores. The results showed that after exposure to simulated space vacuum for 10 days, viability of spores of two *Bacillus* species was reduced up to 35%, but all spores retained their large Ca^2+^-dipicolinic acid depot. Some of the killed spores did not germinate, and the remaining germinated but did not proceed to vegetative growth. The vacuum treatment slowed spore germination, and changed average times of all major germination events. In addition, viable vacuum-treated spores exhibited much greater sensitivity than untreated spores to dry heat and hyperosmotic stress. Among spores’ resistance mechanisms to high vacuum, DNA-protective α/β−type small acid-soluble proteins, and non-homologous end joining and base excision repair of DNA played the most important roles, especially against multiple cycles of vacuum treatment. Overall, these results give new insight into individual spore’s responses to space vacuum and provide new techniques for microorganism analysis at the single-cell level.

## Introduction

Can any terrestrial life survive in outer space? This question has long been of great interest in astrobiology because of the mystery of the origin of Earth’s life and the dispersion of life in Universe.^1,2^ Outer space is an extremely challenging environment for all forms of terrestrial life, and is characterized by high vacuum, an intense radiation field of galactic and solar origin, extreme temperatures, and microgravity.^1,2^ Among these characteristics, water desorption due to high vacuum (10^−14^–10^−4^ Pa) is particularly notable, since water is one of the principal ingredients of cellular life and some water activity is indispensable for organismal growth.^3^ Thus the high vacuum conditions of outer space present a major challenge for any form of life, and only microorganisms in a dormant and resistant state might survive in outer space. That is why bacterial spores with their dormancy and extremely high resistance to harsh conditions are frequently used in astrobiology.^4–9^ One of the interests in astrobiology is whether living organisms can be transported between the planets of the Solar System by mechanisms such as on meteorites, and, if so, the surviving organisms must be highly resistant to the severe strain of a long journey through space.^10,11^ Dormant spores of bacteria are recognized as the hardiest known form of life on Earth and considerable effort has been invested in understanding the molecular mechanisms responsible for the extreme resistance of spores to harsh treatments.^2^ However, whether individual spores undergo severe molecular and morphological changes under space vacuum and how individual spores respond to space vacuum are not clear. It is also unclear whether individual spores exposed to space vacuum during a simulated space journey can return to the life when proper conditions are provided, and how rapidly the vacuum-exposed spores can do this. Consequently, knowledge about individual spore’s responses to space conditions will help identify specific effects of space conditions on spores and address the question of possible transport of life through space as spores.^12^

Bacterial spores are formed in sporulation by many Gram-positive Bacilli and Clostridia, and are metabolically dormant.^12–15^ The return to life of such spores in germination has been the subject of significant research interest for four major reasons: (i) novel regulatory systems allow spores to remain in a dormant state for years and yet return to vegetative growth through germination and then outgrowth in minutes; (ii) spores have extremely high resistance to a wide variety of agents including high temperatures, desiccation, radiation, and toxic chemicals; (iii) spores of some species cause food spoilage and foodborne disease, as well as some serious human diseases, but after germination they lose their resistance properties and become relatively easy to kill;^16–18^ and (iv) spores have long been used as experimental organisms for astrobiology research, as their extreme resistance may allow them to survive in the harsh environment of outer space.^19^

Many studies have found that spores exposed to various simulated space environments or in space when carried by balloons, rockets, or spacecraft can remain viable to some extent, provided they are shielded against the intense solar ultraviolet radiation.^1,2,5,6^ For example, it was found that wide-type *B. subtilis* spores survive up to ~70% after 10 days in space vaccum and there was still 1–2% viable spores after nearly six years in space.^6^ However, the germination and outgrowth of, and subsequent growth from, individual spores exposed to simulated space vacuum have not been studied. Notably, many simulated space parameters are used by the food industry to inactivate spores^20,21^ and vacuum treatment may be one method to either prevent spore germination and subsequent risk or accelerate spore germination in order to facilitate destruction of the much less resistant germinated spores.

Spore germination involves complex signal transduction pathways and biophysical events that have been studied best in spores of *B. subtilis*.^14,22,23^ Germination of *B. subtilis* spores can be triggered by L-alanine or L-valine or a combination of L-asparagine, D-glucose, D-fructose, and K^+^ (AGFK) by interacting with specific nutrient germinant receptors (GRs) in the spore’s inner membrane (IM), the GerA GR responding to L-alanine/L-valine, and the GerB and GerK GRs responding to the AGFK mixture.^14,18^ There are also GR-independent germinants, including high hydrostatic pressure, a 1:1 chelate of Ca^2+^ with dipicolinic acid (CaDPA), and cationic surfactants, such as dodecylamine.^14,22,23^ During *B. subtilis* spore germination, several major events occur in a defined order. The earliest measurable event after germinant-GR interaction is termed commitment, in which even if the germinant is removed, committed spores continue through germination.^23^ Commitment is followed by release of monovalent cations (H^+^, Na^+^, and K^+^), as well as the spore core’s large pool (~25% of spore core dry weight) of CaDPA, and its replacement by water. CaDPA release then triggers cortex-lytic enzymes to degrade spores’ peptidoglycan (PG) cortex, and completion of cortex PG degradation allows the swelling and further hydration of the spore core. Once the core water content has increased to ~80% of wet weight, equal to that in the growing cell, metabolism in the core begins, followed by macromolecular synthesis, spore elongation, and growth in the processes of outgrowth and then vegetative growth.

Normally, the process of an individual spore’s germination is divided into four phases according to a spore’s optical intensity in phase-contrast or differential interference contrast (DIC) microscopy, with the different phases begining or ending at times *T*_1_, *T*_lag_, *T*_release_, and *T*_lys_.^23–25^ *T*_1_, also referred as *T*_leakage_, is the time when slow CaDPA leakage begins after germinant addition and is probably coincident with the time of commitment. *T*_lag_ is the time when the initiation of very rapid CaDPA release begins after the start of slow CaDPA leakage, and *T*_release_ is the time for completion of rapid CaDPA release. Following *T*_release_, there is a further small decline in spore refractility due to the hydrolysis of the spore cortex PG and spore core swelling and hydration; the time when spore refractility becomes constant is termed *T*_lys_. These parameters, as well as Δ*T*_leakage_ = *T*_lag_ − *T*_1_, Δ*T*_release_ = *T*_release_ − *T*_lag_ and Δ*T*_lys_ = *T*_lys_ − *T*_release_, are all major kinetic parameters of spore germination. After cortex PG hydrolysis, spores undergo outgrowth in which RNA, protein, and finally DNA are synthesized. As shown in this work, during this period, spores’ length slowly increases to ~1.5-fold of that of dormant spores, and then the outgrowing spore emerges from the spore coat; at this time spore length decreases slightly. This latter time point is now designated as *T*_elong_.

Experiments using simulated space vacuum and in space have shown that high vacuum treatment for 1–10 days results in significant spore inactivation.^1,5,6^ Previous work has shown that the crucial event leading to spore inactivation by high vacuum is water desorption, which exerts a strong mechanical stress on the spore envelope, cortex, and membranes, and affects the structural integrity of macromolecules, such as lipids, proteins, and, probably most importantly, nucleic acids.^7,26,27^ However, individual spore’s responses to space vacuum are unclear and several obvious questions about high vacuum-treated spores warrant further investigation, including: (i) do vacuum-killed spores, which cannot proceed to vegetative growth after high vacuum treatment, retain CaDPA; (ii) if they retain CaDPA, do vacuum-killed spores germinate when assessed by release of CaDPA and full hydration of the spore core; (iii) do germinated high vacuum-killed spores progress into outgrowth or cell elongation and cell division; (iv) is the germination, outgrowth, and vegetative growth of high vacuum-treated spores decreased and/or slowed as high vacuum treatment is extended; (v) are resistance properties of spores surviving high vacuum treatment altered; and (vi) what spore features and components are most important in spore survival in high vacuum? In the current work, the viability and the kinetics of germination, outgrowth, elongation, and subsequent growth of and from hundreds of individual spores of *Bacillus* species exposed to simulated space vacuum (hereafter high vacuum) were investigated using Raman spectroscopy and live-cell differential interference contrast (DIC) microscopy. The effects of dry heat or high osmolarity on spores surviving high vacuum treatment was also examined. Finally, the roles of various spore protective components and DNA repair pathways in spore resistance to high vacuum were also determined. Taken together, the results of this study provide new insight into space vacuum effects on bacterial spores.

## Results

### Spore viability and CaDPA content after high vacuum exposure

**Fig. 1:**
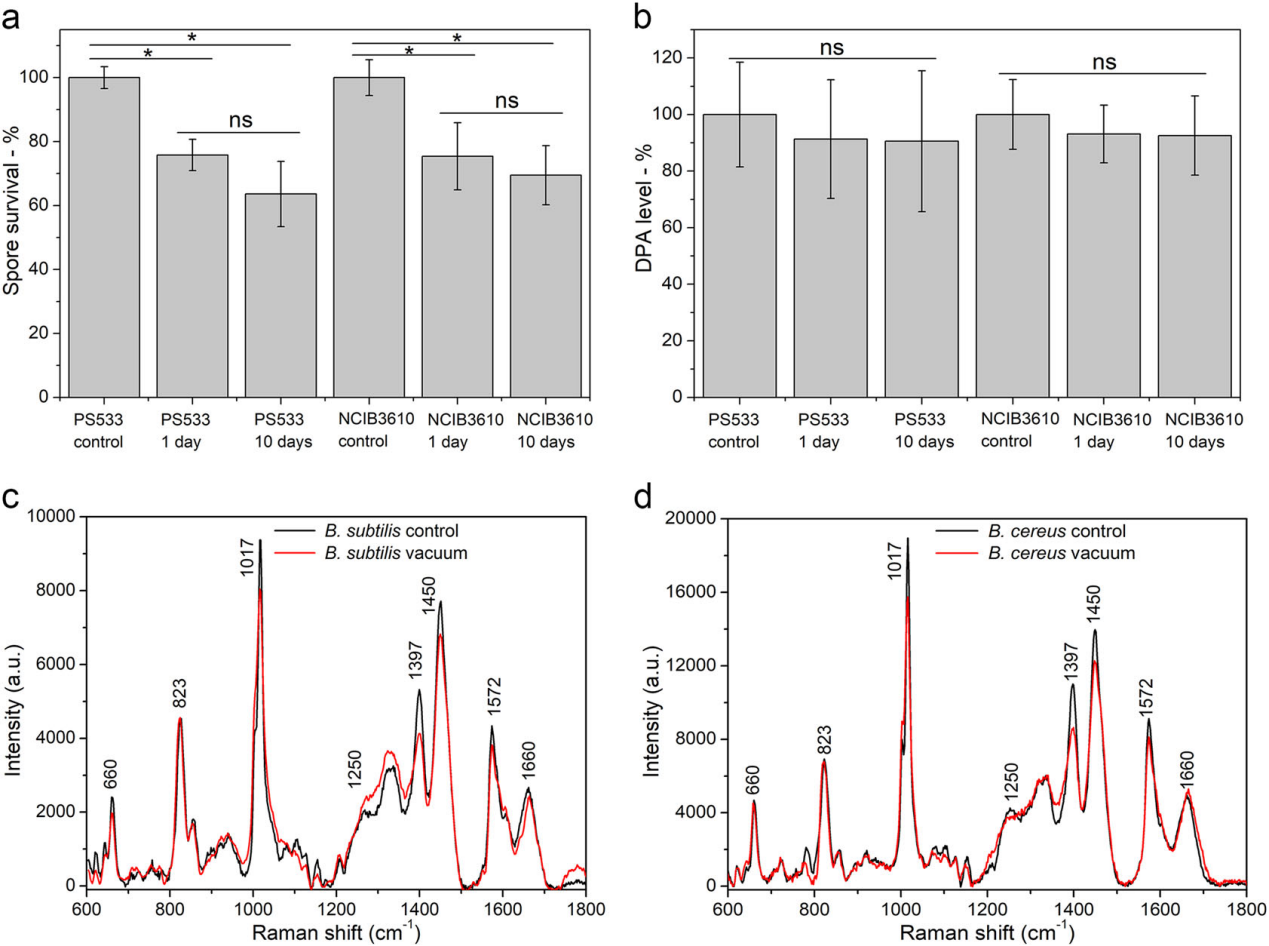
Viability, DPA levels and Raman spectra of spores with and without high vacuum treatment. **a**, **b** Viability of *B. subtilis* PS533 and NCIB 3610 spores that were exposed to high vacuum (<2.6 × 10^−5^ Pa) for various periods. Spores were first dried in a centrifuge tube and were exposed to high vacuum for 1 or 10 days. Spores that were dried in ambient laboratory pressure but not exposed to high vacuum are the control spores. After high vacuum exposure, spores were suspended in 100 uL distilled water and cultured on LB plates at 37 °C, and spore viability was normalized to that of the control spores. **c**, **d** Average Raman spectra of multiple single spores of *B. subtilis* PS832 **c** and *B. cereus* **d** at atmospheric pressure (1.0 × 10^5^ Pa) (control) and high vacuum. The spectra of spores during the exposure to high vacuum were directly measured using image-guided micro-Raman spectroscopy. The laser power was 20 mW and integration time was 30 s, and spectra shown were averaged over 30 individual spores. *P < 0.05%; ns, no significance

After exposure to high vacuum for 1 or 10 days, the viability of *B. subtilis* PS533 (wild type) spores fell 25 and 36%, respectively, compared to that of untreated spores (Fig. 1a). While the difference in viability between vacuum-treated and untreated spores was significant, the difference in spore viability after 1 and 10 days of vacuum exposure was not (Fig. 1a). A similar loss in viability after high vacuum exposure was also obtained with spores of another wild-type *B. subtilis* strain NCIB 3610 (Fig. 1a).

To observe the effects of high vacuum treatment on spore properties, laser tweezers Raman spectroscopy (LTRS) was used to compare the Raman spectra of *B. subtilis* spores given high vacuum treatment for 1 or 10 days and then suspended in water. The results showed that there were no obvious Raman spectral changes in spores of *B. subtilis* PS533 and NCIB 3610 suspended in water after high vacuum treatment, and spores of both strains had similar CaDPA levels before and after high vacuum treatment (Fig. 1b). If multiple individual *B. subtilis* spores were directly examined under high vacuum by image-guided micro-Raman spectroscopy (Supplementary Fig. 1), all spores exposed to high vacuum for 1 day retained their CaDPA as expected. However, unexpectedly, intensities of Raman bands of CaDPA at 1017, 1397, 1450, and 1572 cm^−1^ were all slightly reduced, while the Raman band intensity at 1660 cm^−1^ due to protein was relatively unchanged, indicating that: (i) the spore core environment around CaDPA was changed under high vacuum (Fig. 1c) and (ii) there was minimal protein denaturation by high vacuum treatment. A similar result was obtained for *B. cereus* spores (Fig. 1d). Importantly, when high vacuum-treated spores were suspended in water, the Raman spectra of their CaDPA reverted to that of control spores.

### Nutrient and non-nutrient germination of untreated and high vacuum-treated B. subtilis spores

**Fig. 2:**
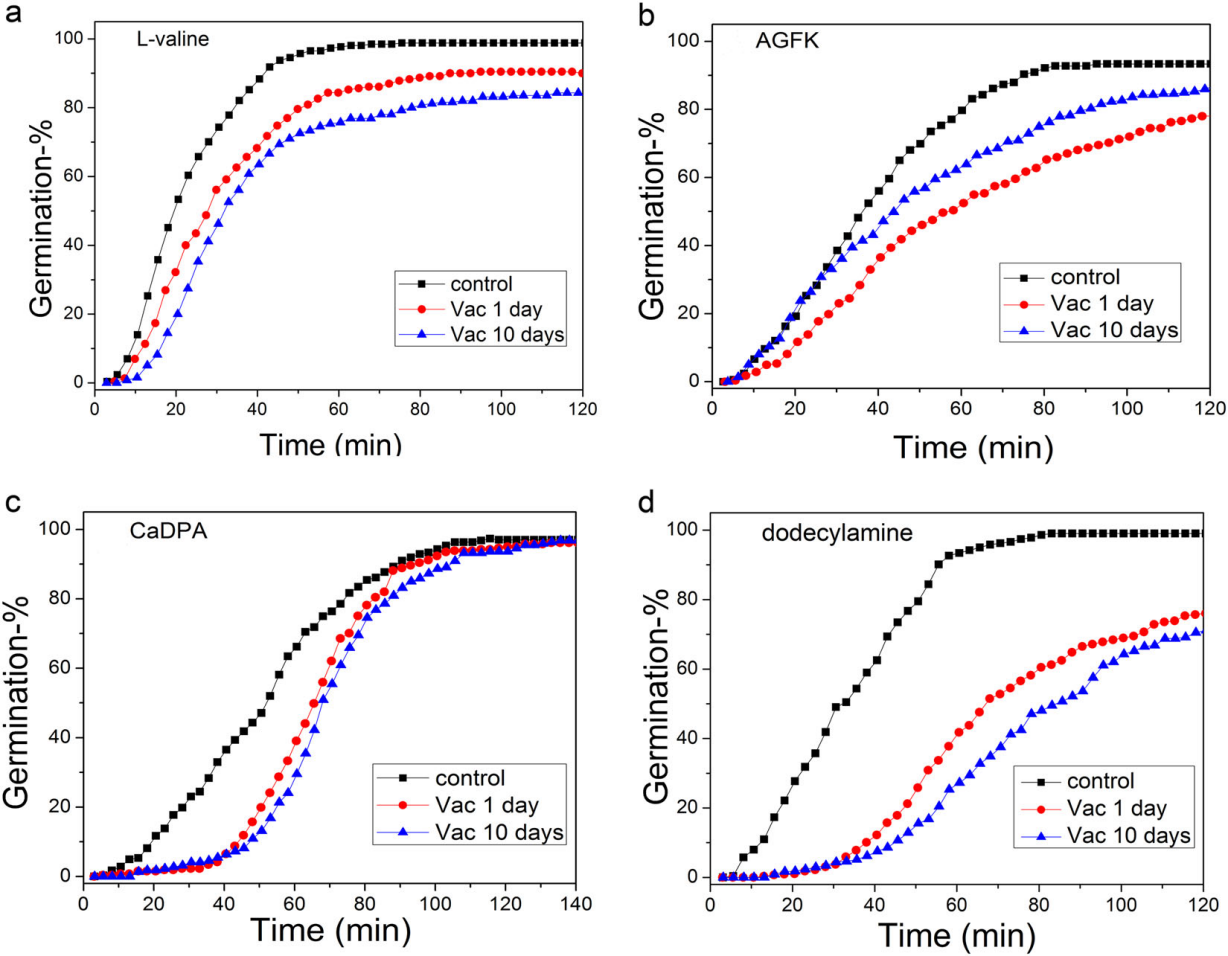
Germination of *Bacillus subtilis* PS533 spores with and without high vacuum treatment. Spores without or with high vacuum treatment (<2.6 × 10^−5^ Pa) for 1 or 10 days were germinated with 1 mM L-valine in 25 mM K-Hepes (pH 7.4) at 37 °C **a**, 10 mM AGFK in 25 mM K-Hepes (pH 7.4) at 37 °C **b**, 60 mM CaDPA at 25 °C **c**, and 1.0 mM dodecylamine at 50 °C **d**, and germination of 500 individual spores was assessed, all as described in Methods

Germination of *B. subtilis* spores can be triggered by GR-dependent nutrient germinants such as L-valine or AGFK or GR-independent non-nutrient germinants, such as exogenous CaDPA or dodecylamine. In order to investigate the damage caused by high vacuum exposure to spores’ germination, the germination of spore populations with all germinants noted above was examined. The results showed that high vacuum treatment for 1 or 10 days decreased rates of germination with all germinants, and decreased the percentages of germination with all germinants except CaDPA (Fig. 2a–d).

While the analysis of spore populations provided some information on the germination behavior of high vacuum-treated spores, these germination curves were the average behavior of many different spores whose individual behavior could differ due to intrinsic spore germination heterogeneity.^14,22,23^ Therefore, germination of multiple individual untreated and high vacuum-treated *B. subtilis* PS533 spores was also examined with the germinants mentioned above (Table 1; Supplementary Figs 2-5). Individual untreated spores germinated rapidly, most by ~60 min (Fig. 2), and the rapid CaDPA release for individual spores between *T*_lag_ and *T*_release_ took place almost in parallel (Supplementary Fig. 2a-5a); this was also true for spores high vacuum treated for 1 or 10 days (Supplementary Figs 2b,c-5b,c). However, the germination kinetics of multiple individual high vacuum-treated spores differed significantly from those of untreated spores and there were differences in the effects on germination with different germinants. In particular, with GR-dependent germinants, the average values of *T*_1_, *T*_lag_, *T*_release_, and Δ*T*_lys_ increased with high vacuum exposure, but Δ*T*_leakage_ and Δ*T*_release_ values were changed minimally (Table 1). With the non-nutrient germinants CaDPA and dodecylamine, the results were more complex. Average values of *T*_1_ and *T*_lag_ in CaDPA and dodecylamine germination increased after high vacuum exposure. However, with CaDPA germination, as high vacuum exposure time increased, the Δ*T*_release_ times decreased, and the Δ*T*_lys_ times increased while Δ*T*_leakage_ times did not change. In contrast, with dodecylamine germination, as high vacuum exposure time increased, values of Δ*T*_leakage_ and Δ*T*_release_ values increased and those of Δ*T*_lys_ decreased (Table 1).

**Table 1:** Mean values and standard deviations of *T*_1_, *T*_lag_, *T*_release_, and Δ*T*_release_ for germination of *B. subtilis* PS533 untreated and high vacuum-treated spores with different germinants^a^

Treatment conditions	No. of spores examined (% spore germination)	*T*_1_ (min)	*T*_lag_ (min)	Δ*T*_leakage_ (min)	Δ*T*_release_ (min)	Δ*T*_lys_ (min)
1 mM L-valine (untreated)	449 (99%)	21.8 ± 19.2	23.0 ± 19.5	1.3 ± 0.9	5.0 ± 1.4	12.7 ± 4.5
1 mM L-valine vac 1 day	467 (91%)	26.5 ± 19.0	28.1 ± 17.6	1.6 ± 1.9	4.8 ± 1.8	16.1 ± 5.1
1 mM L-valine vac 10 day	397 (84%)	27.9 ± 17.1	29.6 ± 17.1	1.5 ± 1.8	4.9 ± 1.4	17.8 ± 9.5
AGFK (untreated)	402 (93%)	41.5 ± 21.1	42.5 ± 21.2	1.0 ± 0.5	4.6 ± 1.1	11.0 ± 4.3
10 mM AGFK vac 1 day	416 (85%)	43.5 ± 28.0	45.4 ± 28.2	2.0 ± 1.6	4.7 ± 1.0	15.0 ± 13.3
10 mM AGFK vac 10 day	456 (78%)	43.5 ± 26.2	45.0 ± 26.4	1.5 ± 1.0	4.5 ± 1.1	18.3 ± 6.5
CaDPA (untreated)	299 (97%)	30.4 ± 13.6	58.4 ± 20.0	28.0 ± 13.5	4.9 ± 1.3	25.5 ± 8.6
CaDPA vac 1 day	437 (97%)	35.5 ± 14.2	63.3 ± 17.5	27.8 ± 13.4	4.6 ± 1.6	26.4 ± 11.4
CaDPA vac 10 day	464 (97%)	39.4 ± 17.8	66.0 ± 21.2	26.6 ± 12.3	3.8 ± 1.3	29.6 ± 9.5
Dodecylamine (untreated)	414 (99%)	35.1 ± 23.3	37.2 ± 22.7	2.1 ± 2.8	4.1 ± ± 1.4	20.6 ± 18.7
Dodecylamine vac 1 day	447 (87%)	42.8 ± 25.2	49.8 ± 32.9	7.0 ± 13.2	4.6 ± 1.2	15.9 ± 12.4
Dodecylamine vac 10 day	367 (82%)	60.2 ± 34.8	72.6 ± 37.0	12.4 ± 15.5	4.7 ± 1.3	16.0 ± 9.7

*vac* vacuum exposure

^a^PS533 (wild type) spores with or without high vacuum treatment were germinated for 2–2.5 h with various germinants, and kinetic parameters of spore germination were determined as described in Methods and in Supplementary Figs 2–5. All values shown are from ~100 individual spores that contained CaDPA and germinated

### Comparison of germination, outgrowth, elongation, and subsequent growth from untreated and high vacuum-treated B. subtilis spores

**Fig. 3:**
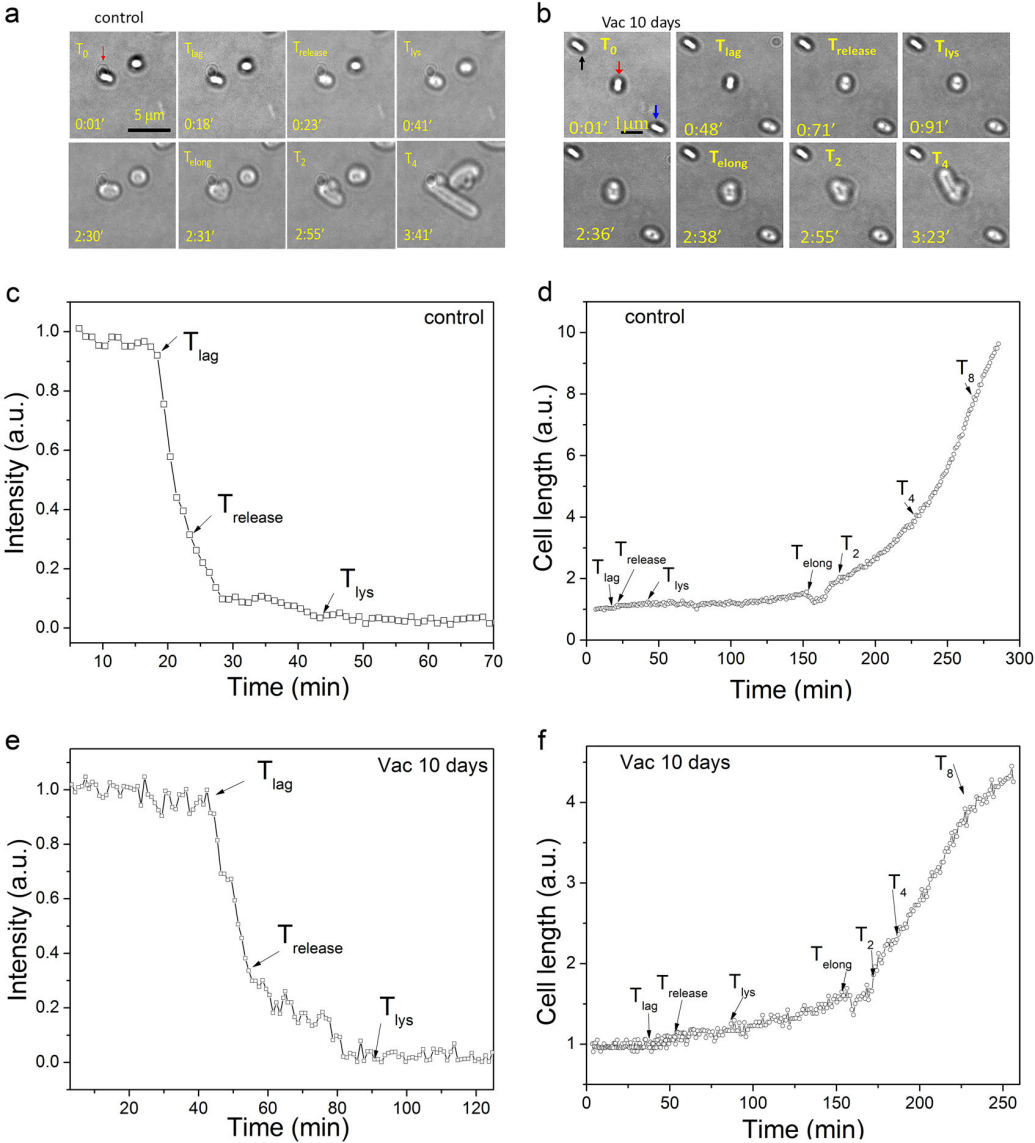
Germination, outgrowth, elongation, and growth of individual PS533 spores without or with high vacuum treatment. Spores with or without a 10 day high vacuum treatment were incubated on an LB medium agar pad on a microscope coverslip at 37 °C and spore and cell images were obtained by DIC microscopy as described in Methods. **a**, **b** Cell images at various times. In the 0.01 min images, the red arrows in a and b indicate spores that germinated and ultimately grew, the black arrow in b indicates a spore that never germinated, and the blue arrow in (**b**) indicates a spore which germinated but did not grow. **c**, **e** Intensities of bright-field DIC images at various culture times; and **d**, **f** spore/cell length vs culture time

In order to analyze the effects of high vacuum treatment on spore germination, outgrowth, elongation, and subsequent growth, spores of *B*. *subtilis* PS533 with and without high vacuum treatment were cultured on an LB medium agar pad on a microscope coverslip at 37 °C, and DIC images were recorded every 60 s (Fig. 3a, b). The results analyzing a single spore showed that when the untreated spore began rapid CaDPA release at *T*_lag_, spore length increased slowly. Between the *T*_lys_ and *T*_elong_ time points, this spore initiated outgrowth, and spore length slowly increased ~1.5-fold over that of the dormant spore. The new cell then emerged from the original spore coat, spore length decreased slightly at T_elong_ and, then cell length began increasing rapidly leading to the generation of a growing cell at *T*_2_ (Fig. 3a, c, d; and see also Supplementary Movie 1). Perhaps the emergence of the outgrowing spore from the spore coat escapes the constraint of the coat on any increase in spore width. Similar phenomena were observed for germination, outgrowth, elongation, and subsequent growth of a single high vacuum-treated spore (Fig. 3b, e, f; and see Supplementary Movie 2). However, a number of high vacuum-treated spores germinated but did not undergo outgrowth and elongation, and did not give rise to growing cells (Fig. 3b). Presumably these are spores that were killed by the high vacuum treatment.

**Fig. 4:**
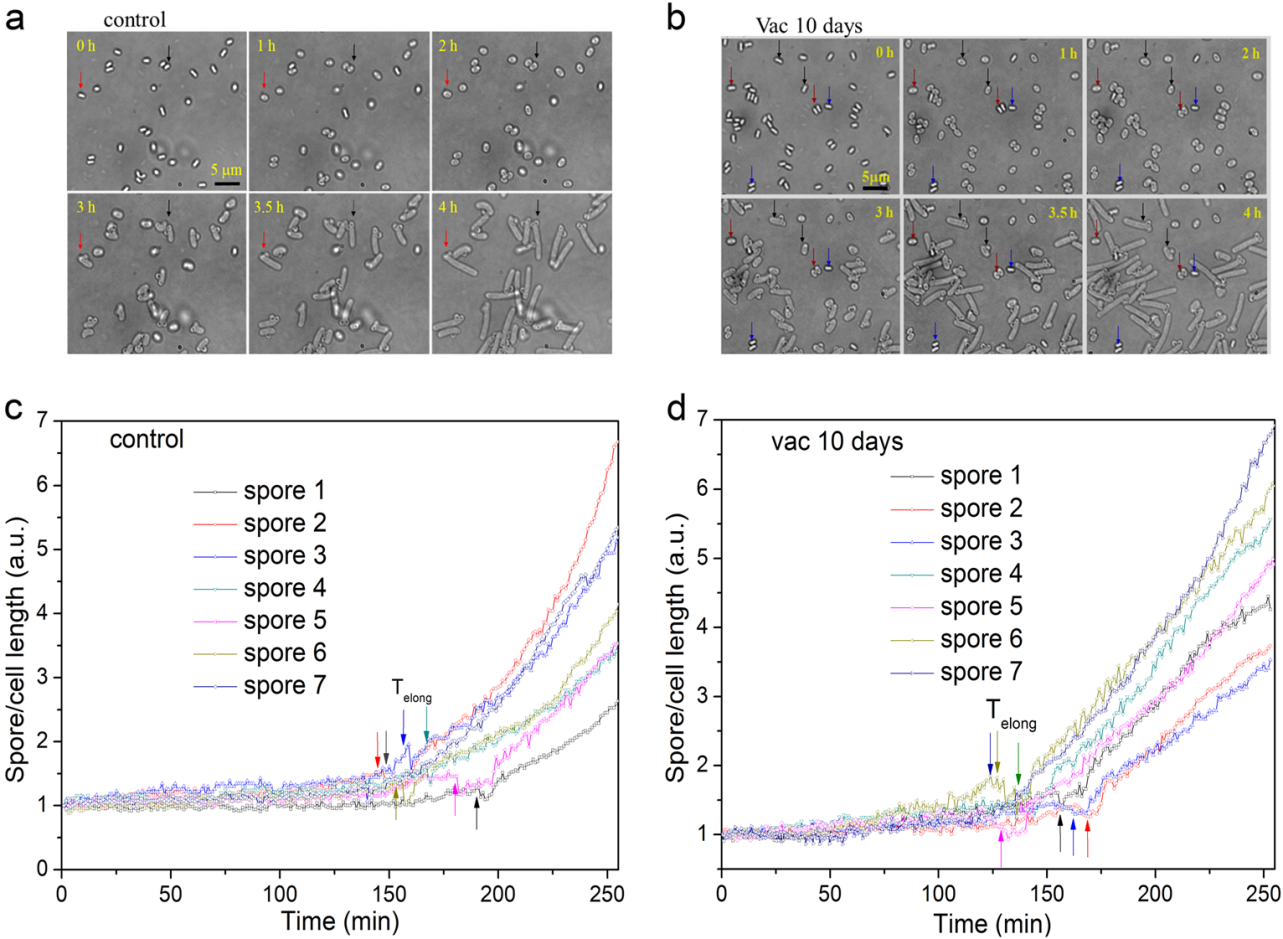
Germination, outgrowth, elongation, and growth of multiple individual untreated *B. subtilis* PS533 spores without **a**, **c** or with **b**, **d** a 10 day high vacuum treatment. Untreated or high vacuum-treated spores were cultured on an LB medium agar pad on a microscope coverslip at 37 °C and bright-field images were recorded every 60 s for 4 h as described in Methods. **a**, **b** Bright-field images of individual spores at various culture times. In a panels the red and black arrows show images of two spores that germinated and grew; in panel **b** the blue, red, and black arrows show the images of spores that never germinated, germinated but did not grow, and germinated and grew, respectively. **c**, **d** Cell length vs time for 7 individual untreated and high vacuum-treated spores that germinated and grew, and arrows denote the T_elong_ times

Analysis of the germination, outgrowth, elongation, and growth kinetics of multiple individual spores using phase-contrast microscopy gave similar results to those seen by DIC microscopy (Fig. 4a, b). Furthermore, comparison of the spore/cell length vs time curves of multiple germinated and growing spores without or with high vacuum exposure found that rates of cell length increase following T_2_ exhibited no significant difference (compare Fig. 4c, d). However, while 34/35 of untreated spores germinated and grew by 240 min, of the 57 high vacuum-treated spores observed, 4 did not germinate, and 8 germinated but did not initiate outgrowth and subsequent elongation and growth by 240 min (Fig. 4c, d). Thus 21% of high vacuum-treated spores appeared to be dead, a percentage not too different from that seen when viability of untreated and high vacuum-treated spores was compared (Fig. 2a).

### Changes in spore stress resistance after high vacuum treatment

**Fig. 5:**
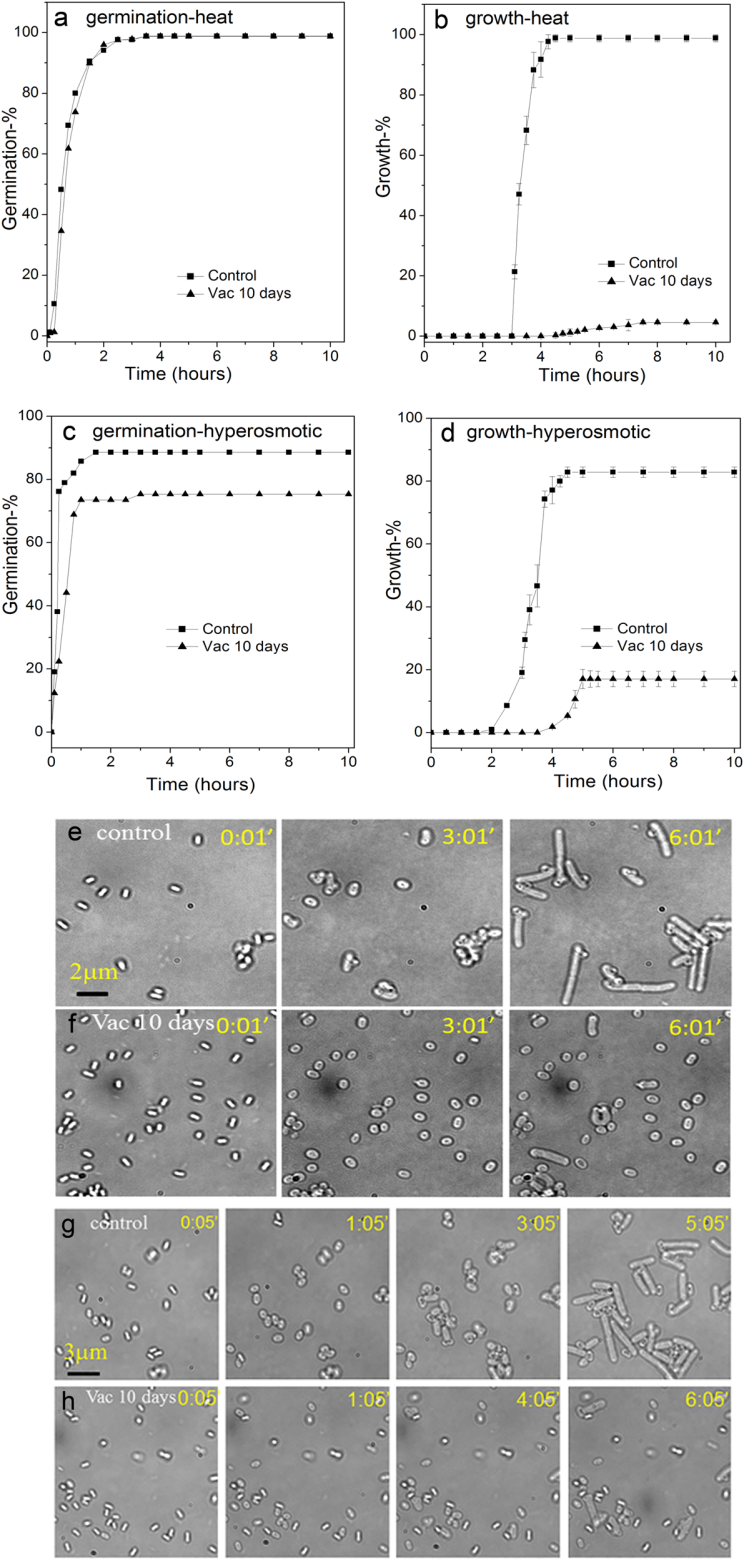
Changes in spore resistance to dry heat or hyperosmotic stress after high vacuum treatment. The percentages of germination **a**, **c** and growth **b**, **d** of multiple individual untreated (black box) or 10 day high vacuum-treated (black triangle) *B. subtilis* PS533 spores which were then treated with dry heat at 140 °C for 7 min **a**, **b** and incubated at 37 °C on either an LB medium agar pad, or not treated with dry heat but incubated at 37 °C on an LB medium agar pad with 1 M NaCl **c**, **d**; the germination and growth of > 300 individual spores was followed, all as described in Methods. Bright-field images were recorded every 60 s for ~6 h for dry heat-treated **e**, **f** and hyperosmotic cultures **g**, **h**

Previous work has shown that spores surviving a number of inactivation treatments may be damaged and less able to resist an additional stress such as high heat or high salt levels in recovery media.^26,28^ Notably, the temperature in outer space may change from very low to very high depending on the orientation to the sun and the albedo of the spacecraft, and this might impact spore survival. To examine the combined effects of high vacuum and high heat, untreated and high vacuum-treated spores were exposed to dry heat at 140 °C for 7 min. Analysis of these treated spores’ germination and growth on an LB medium agar pad showed that the combination of high vacuum and dry heat did not affect spore germination appreciably (Fig. 5a, e). However, only ~4% of the spores given both treatments exhibited growth (Fig. 5b, f). In contrast, 64% of spores given high vacuum treatment alone grew (Fig. 1a), while growth from untreated spores given the dry heat treatment was unaffected (Fig. 5b, e).

To further examine the resistance properties of spores surviving high vacuum exposure, germination, and growth of untreated and vacuum-treated spores was determined on an LB medium agar pad with or without 1 M NaCl. The results showed that germination of high vacuum-treated spores was reduced only slightly on the high salt medium (Fig. 5c, g). While the growth of the untreated spores on the high salt medium was reduced to 83%, the growth from high vacuum-treated spores was reduced to 17% on the high salt medium (Fig. 5d, h), in contrast to 64% of high vacuum-treated spores grew on low salt medium (Fig. 1a). This ~4-fold reduction in growth indicates that the high vacuum-treated spores are more sensitive to hyperosmotic stress. This salt sensitivity is consistent with damage to IM proteins or the IM itself by high vacuum.^28^ Taken together, the results indicate that the combination of high vacuum with either dry heat or hyperosmotic stress caused synergistic spore killing.

### Spore structural properties and DNA repair involved in spore survival under high vacuum

**Fig. 6:**
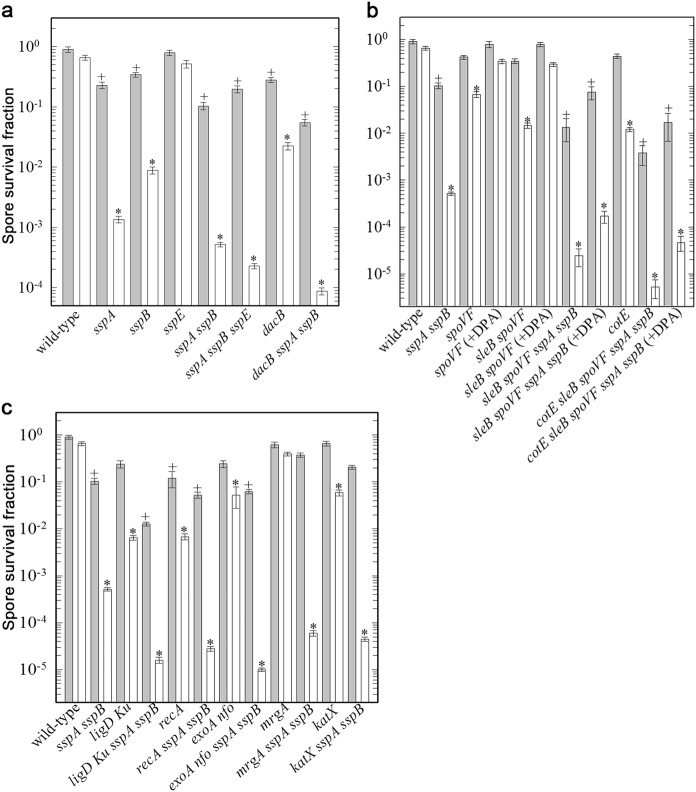
High vacuum killing of *B. subtilis* spores with defects in spore structural components **a**, **b** or DNA repair (**c**). Spores of strain PS832 and its isogenic derivatives were exposed to high vacuum and spore survival was determined as described in Methods. The spore surviving fraction was determined from the ratio of N/N_0_, with N_0_ the number of CFU of the untreated spores (spores stored dry in a laboratory desiccator) and N the high vacuum-treated spores. Values were analyzed in multigroup pairwise combinations, and differences with *P* values of <0.05 were considered statistically significant. Gray bars, laboratory storage; white bars, high vacuum-treated spores. Plus, spore resistance to laboratory storage significantly different than the wild type; astereisk, spore resistance to high vacuum significantly different than the wild type

Previous work has shown that high vacuum kills spores at least in part by DNA damage.^2^ In order to determine the significance of the factors protecting spores from high vacuum damage, possible spore protective factors, including different spore structures, core water, and DPA content, as well as DNA repair mechanisms, were systematically investigated using spores with alterations in these various factors (Fig. 6a–c). This work showed that protection by the two major DNA-binding α/β-type SASPs (SspA or SspB) played the most important role in protecting spores against high vacuum, with spores lacking both major proteins of this type, termed α^−^β^−^ spores, killed ≥99.9% by one high vacuum treatment. Indeed, even desiccation alone reduced α^−^β^−^ spore viability ~10-fold as expected.^2,18^ However, the other major SASP, SspE, which is not bound to spore DNA, played a minimal role at best in spore DNA protection against desiccation and high vacuum. Other genes in which mutations decreased spore resistance to high vacuum included *dacB*, mutation of which alters spore cortex structure and gives spores with an elevated core water content; *cotE*, mutation of which eliminates assembly of the spore outer coat; and *sleB spoVF* mutations which give DPA-less spores, which are stable and have an elevated core water content; when this strain is sporulated with exogenous DPA, developing spores can take this up to levels close to that in wild-type spores.^29^ Notably, the decreased high vacuum resistance of *sleB spoVF* spores alone or with other mutations was increased significantly when *sleB spoVF* strains were sporulated with DPA. Combination of *sspA sspB* mutations with *dacB, sleB spoVF*, or *cotE* mutations further reduced spore high vacuum resistance. Overall, the relative importance of various protective factors in spore high vacuum resistance was α/β-type SASP » core water content (*dacB*) >outer coat layer (*cotE*) ≈ spore DPA content (*sleB spoVF)* (Fig. 6a–c).

With regards to the role of individual DNA repair pathways, Nfo and ExoA, apurinic/apyrimidinic endonucleases involved in the base excision repair pathway (BER), displayed the most significant role, and the role of BER seemed greater than either homologous recombination repair mediated by RecA or non-homologous end joining (NHEJ) mediated by LigD Ku (Fig. 6c). The latter data suggest that the spectrum of high vacuum lesions in spore DNA includes more abasic sites than DNA strand breaks.^19,30–32^ Loss of the spore-specific catalase KatX had only a small effect on spore high vacuum resistance, and loss of the DNA-binding stress protein MrgA had no effect (Fig. 6c).

**Fig. 7:**
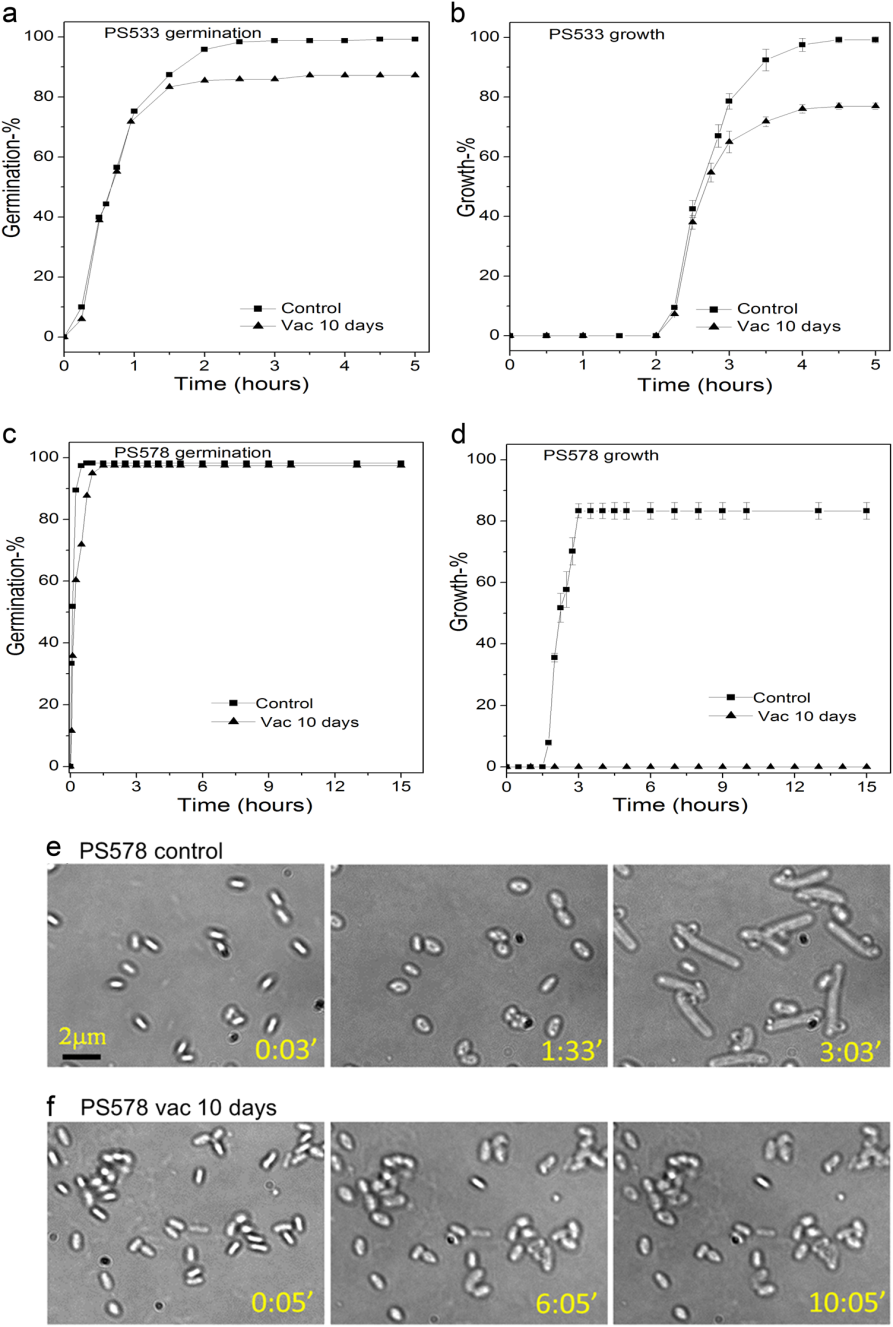
Germination **a**, **c** and growth **b**, **d** of multiple individual untreated or high vacuum-treated *B. subtilis* PS533 (wild type) **a**, **b** or PS578 (α^−^β^−^) **c**–**f** spores. *B. subtilis* PS533 or PS578 spores that were untreated (black square) or high vacuum-treated for 10 days (black triangle) were incubated on an LB medium agar pad at 37 °C and the percentages of germination and growth of ~500 individual spores was determined, all as described in methods. Sequential bright-field images of individual PS578 spores without **e** or with **f** a 10-day high vacuum exposure were also obtained at various times

In order to further analyze the effects of the most important factor in spore protection against high vacuum, the major α/β-type SASP, we examined the germination and outgrowth of individual α^−^β^−^ spores given a high vacuum treatment (Fig. 7a, b). As noted above, the germination and growth of wild-type *B*. *subtilis* spores given a 10 day high vacuum treatment were reduced 23% and 33%, respectively, compared to that of untreated spores (Fig. 7a, b). Notably, α^−^β^−^ spore germination was minimally affected if at all by a 1 day high vacuum treatment, but none of these treated spores grew among 1000 observed spores (Fig. 7c, d and data not shown).

### Effects of multiple cycles of high vacuum treatment on spore survival

**Fig. 8:**
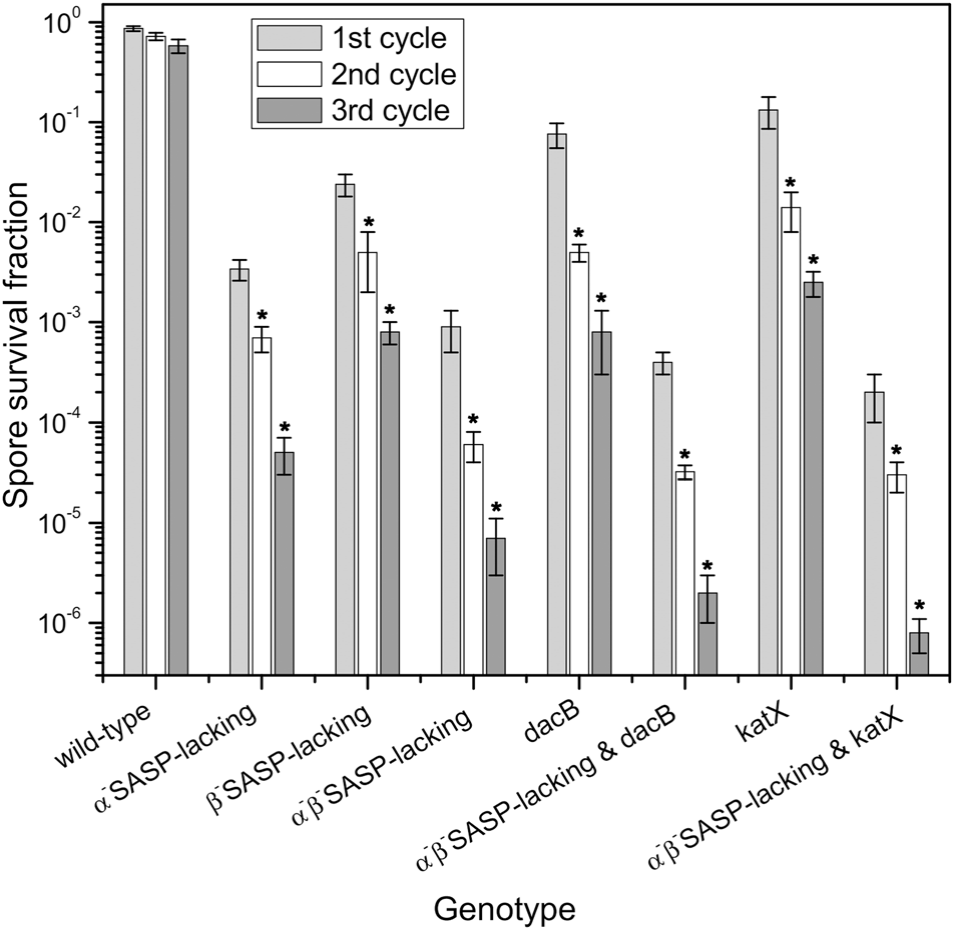
Killing of *B. subtilis* spores of different mutants by multiple cycles of high vacuum treatment. *B. subtilis* spores of different isogenic strains were exposed to high vacuum (~10^−6^ Pa) for 24 h, followed by return to atmospheric pressure for ~30 min before the next cycle of high vacuum treatment. Samples were ~10^8^ spores immobilized on stainless steel discs, and CFU before and after each treatment were determined relative to CFU for control samples dried under ambient laboratory conditions. All experiments were conducted in triplicate, and standard deviations for all data are shown

While only limited inactivation (20–35%) of spores of two wild-type *Bacillus* strains was obtained by 1–10 days of high vacuum treatment (Fig. 1), this was increased to ~40% by three cycles of high vacuum and then atmospheric pressure (Fig. 8). Higher inactivation (~80%) was observed if spores were fully hydrated between high vacuum treatments (Supplementary Table 1). Given the significant killing of spores lacking DNA protection capacity, we also examined the killing of α^−^β^−^ *dacΒ* and *katX* spores by multiple cycles of high vacuum treatment (Fig. 8). These experiments showed that two cycles of high vacuum treatment caused significantly more spore killing compared to one cycle, with three cycles of high vacuum treatment giving further significant killing. In addition, α^−^β^−^ spores with additional defects in core water content (α^−^β^−^*dacB*) or damage protection (α^−^β^−^*katX*) exhibited even more inactivation compared to spores with only single defects. Taken together, the results were consistent with our expectation that DNA protection would play a significant role in defending spores against multiple cycles of high vacuum treatment.

## Discussion

Previous studies have found that when typical laboratory vacuum systems are used for drying or freeze-drying spores, wild-type *Bacillus* spores exhibit no significant killing even under the conditions of prolonged desiccation or multiple cycles of freeze-drying and rehydration.^2^ In contrast, previous work has shown that high vacuum equivalent to space vacuum does give significant spore killing.^2^ Notably, the current work indicated that high vacuum killing of spores appeared to be most dependent of the number of times spores were desiccated in high vacuum, and less so on the actual residence time in high vacuum. This high vacuum had two notable effects on spores: (1) killing some spores; and (2) decreasing some spores’ germination with various germinants. Obvious questions about these effects are how high vacuum treatment: (i) kills spores; and (ii) decreases spore germination. The results obtained in the current work confirm that high vacuum treatment kills spores largely by DNA damage. The evidence for this conclusion is as follows. (1) Spore killing by high vacuum is not by: (i) rupture of spores’ inner membrane permeability barrier, since the dead spores retained CaDPA; (ii) massive protein denaturation, since this was not seen by Raman microspectrscopy, although this by no means rules out selective inactivation of a small number of crucial spore proteins as a cause for spore killing by high vacuum; and (iii) destruction of the ability to germinate, since dead spores still germinated and did not lyse, although the dead germinated spores never grew. (2) The fact that spore killing by high vacuum was greatly increased by a number of mutations that eliminate repair of different DNA lesions and by various pathways, including *recA*, *ligD ku*, and *exoA nfo* strongly suggests that DNA damage is the major mechanism whereby high vacuum kills spores. However, the precise damage generated by high vacuum in spore DNA is not clear, and may include not only single and double strand breaks, but also some base loss as well, all as indicated by the effects of loss of different DNA repair genes on spore high vacuum sensitivity. Presumably, detailed analysis of DNA from high vacuum-killed spores could determine the types and amounts of specific damage.

The results of experiments examining effects of mutations in other than DNA repair genes on spore high vacuum sensitivity strongly indicated that the α/β-type SASP, either SASP-β, more so SASP-α, and most of all both of these two major α/β-type SASP played the most important role in high vacuum resistance of spores. This is consistent with the known protection of spore DNA by α/β-type SASP against damage by wet and dry heat, desiccation, oxidative damage, and UV and γ-radiation.^2,33^ While α/β-type SASP play an important role in protection of spore DNA, and thus spore viability against high vacuum treatment, all the dead SASP-less spores germinated relatively normally, strongly suggesting that normal DNA function is not needed for completion of spore germination, including cortex PG hydrolysis. However, a number of other mutations affecting spore properties decreased spore resistance to high vacuum. Two, *dacB* or *sleB spoVF* mutations, result in spores with elevated core water content, the *sleB spoVF* double mutation by eliminating CaDPA in the spore, and the effects of this double mutation were almost eliminated by its sporulation with DPA which almost certainly reduced these spores core water content to close to that of wild-type spores.^29^ However, it is not clear why an elevated core water content would sensitize spores to high vacuum. One possibility is that while DNA in wild-type spores is all almost certainly in an A-like conformation due to α/β-type SASP binding,^18^ in *dacB* or *sleB spoVF* spores with elevated core water some α/β-type SASP might dissociate from DNA giving B-DNA, and perhaps the effects of complete water removal under high vacuum are more damaging to B-DNA than A-like DNA. Again it might be informative to examine the specific DNA damage in high vacuum-treated *dacB* and *sleB spoVF* spores. In addition, a higher core water content alone might lead to differences in the conformation of specific core molecules like DNA, and thus these moelcules susceptibility to deleterious effects of high vacuum.

There are also some minor decreases in spores’ high vacuum resistance upon loss of the: (i) spores’ outer coat in *cotE* spores; (ii) spore-specific catalase KatX; and (iii) and the oxidative stress resistance DNA-binding protein MrgA. One common characteristic of at least two of these mutations, *cotE* and *katX*, is that they greatly sensitize spores to some oxidizing agents, either as dormant or germinated spores,^18,34^ although the effects of oxidizing agents on germinating *mrgA* spores has not been examined. Perhaps high vacuum treatment, either its imposition or removal, results in generation of oxidizing species that gain access to the dormant spore core in *cotE* spores, or into germinating *katX* spores, and thus can cause lethal damage to DNA or some other spore component. Again, as noted above, it could be informative to examine the DNA damage in high vacuum-killed *cotE and katX* spores.

As seen previously with dry heat- or wet heat-killed spores,^20,35,36^ after high vacuum exposure non-viable spores also retained CaDPA and did not release CaDPA upon suspension in water. These results indicate that high vacuum treatment does minimal damage to spores’ IM permeability barrier that retains CaDPA. Indeed, multiple lines of evidence have shown that high vacuum inactivates spores by water desorption.^7,27^ The rates and extents of germination of high vacuum-treated spores with nutrient or non-nutrient germinants were all decreased somewhat compared to that of untreated spores, except for CaDPA germination. The fact that germination of high vacuum-treated spores via GR-dependent germinants in spores’ IM as well as dodecylamine germination by activation of the SpoVA protein channel for CaDPA release, also in spores’ IM, were slowed only slightly makes it extremely difficult to ascribe these changes to damage in specific spore proteins. This damage could also be in spores’ IM itself where GRs and the SpoVA channel for CaDPA are located or in one or more IM proteins. Indeed, the salt sensitivity of high vacuum-treated spores is consistent with IM damage since spore treatment with oxidizing agents can cause salt sensitivity of outgrowing spores, and these treated spores also become heat sensitive.^28^ However, it is clear that damage to the germination apparatus alone is not how high vacuum kills spores.

Finally, it is notable that there was no detectable outgrowth of high vacuum-killed spores, just as seen recently with dry heat-killed *B. subtilis* spores.^35,36^ This may be significant from an applied perspective, since if high vacuum-killed spores don’t outgrow, they will thus likely not synthesize any proteins. This could be especially important with spores from pathogenic bacteria, such as *B. anthracis* and *Clostrdium botulium*.

## Materials and Methods

### Strains, media, and spore preparation

*B. subtilis* strains used in this study are listed in Table 2. *B. cereus* T was originally obtained from H. O. Halvorson. Spores of *B. subtilis* strains were prepared at 37 °C on double strength Schaeffer’s (2×SG) medium agar plates and *B. cereus* spores were also prepared on plates, all as described previously.^35–37^ All purified spores were stored at 4 °C in water protected from light and were >98% free from growing or sporulating cells, germinated spores and cell debris as was observed by phase-contrast microscopy. In some experiments, *B. subtilis sleB spoVF* spores that make stable DPA-less spores were sporulated on plates with 100 μg/ml DPA. While *sleB spoVF* spores prepared without DPA in the sporulation medium have ≤1% of CaDPA levels of wild-type spores, when this strain is sporulated with 100 mg/ml DPA this gives spores that contain ~85% of the CaDPA level in wild-type spore.^29^

**Table 2:** *B. subtilis* strains used in this study^a^

Strain number	Genotype	Phenotype
NCIB3610	Wild type	Ref. ^31^
PS832	Wild type	Laboratory 168 strain
PS533	Wild type	Ref. ^35^
PS578	*sspA sspB*	α^−^β^−^
PS283	*sspA*	Lacking the α/β-type SASP SspA
PS338	*sspB*	Lacking the α/β-type SASP SspB
PS355	*sspA sspB*	α^−^β^−^
PS483	*sspE*	Lacking the γ-type SASP SspE, γ^−^
PS482	*sspA sspB*	α^−^β^−^γ^−^
PS1899	*dacB*	Increased spore core water content
PS2211	*dacB sspA sspB*	Increased spore core water content, α^−^β^−^
FB122	*sleB spoVF*	No spore CaDPA
FB122 (+DPA)	*sleB spoVF*	Some spore CaDPA (sporulation with DPA)
PS3664	*sleB spoVF sspA sspB*	No spore CaDPA and α^−^β^−^
PS3664 (+DPA)	*sleB spoVF sspA sspB*	Some spore CaDPA (sporulation with DPA), α^−^β^−^
PS3722	*ligD Ku*	Non-homologous DNA end joining defect
PS3751	*ligD Ku sspA sspB*	Non-homologous end DNA joining defect, α^−^β^−^
PERM454	*exoA nfo*	Apurinic/apyrimidinic endonucleases defect
PERM450	*exoA nfo sspA sspB*	Apurinic/apyrimidinic endonucleases defect, α^−^β^−^
PS2496	*mrgA*	DNA-binding stress protein defect
PS2507	*mrgA sspA sspB*	DNA-binding stress protein, α^−^β^−^
PS2558	*katX*	Lacks spore catalase
PS2559	*katX sspA sspA sspB*	Lacks spore catalase, α^−^β^−^
PS2318	*recA*	Homologous recombination, DNA repair defects
PS2319	*recA sspA sspB*	Homologous recombination and DNA repair defects, α^−^β^−^
AD28	*cotE*	Spores lack outer coat
PS3747	*cotE sleB spoVF sspA sspB*	No spore CaDPA, spores lack outer coat, α^−^β^−^
PS3747 (+DPA)	*cotE sleB spoVF sspA sspB*	Some spore CaDPA, no spore outer coat, α^−^β^−^ (sporulated with DPA)

^a^All strains are isogenic with B. subtilis PS533 except NCIB3610

### Image-guided multifocus confocal Raman micro-spectroscopy of individual spores in a high vacuum

To explore possible molecular changes in spores under high vacuum, a special vacuum chamber was developed (Supplementary Fig. 1), which allows direct observation of individual spores under high vacuum using image-guided single-cell confocal Raman micro-spectroscopy.^38,39^ Briefly, the spores (~1 μl of 10^8^ spores/ml in water) were air dried on a quartz coverslip (0.1 mm thick) that was sealed on a window of a vacuum chamber pumped with a Turbo pumping station (Pfeiffer Vacuum, Berlin, Germany). The bright-field or phase-contrast image of multiple individual spores adhered in random positions on the quartz coverslip was recorded with an imaging camera, and then analyzed by a MATLAB program to locate their centroid positions in a field of view. These coordinates were used to drive a pair of galvo-mirrors and steer a single laser beam to illuminate the individual spores located. Confocal micro-Raman spectroscopy of the illuminated spores was recorded by a multichannel charge-coupled device (CCD) spectrometer (Princeton Instruments, PIXIS 400BR).

### High vacuum treatment and measurement of spore viability and CaDPA content

High vacuum (< 2.6×10^−5^ Pa) was routinely achieved in a vacuum chamber (Supplementary Fig. 1) pumped by a Turbo pumping station and a Turbo molecular pump (Pfeiffer Vacuum, Berlin, Germany). Spores (~1 µl of ~10^8^ spores/ml in water) in a centrifuge tube were placed inside the vacuum chamber to simulate space vacuum treatment. It took ~2 min for the vacuum chamber to drop from atmospheric pressure to high vacuum by using an isolation valve. In order to be comparable with previous research,^1,6^ spores were exposed to high vacuum for 1 or 10 days. After the high vacuum treatment, the spores were suspended in distilled water to a concentration of ~10^7^ spores/ml and examined by laser tweezers Raman spectroscopy (LTRS) as described previously.^40–42^

To investigate effects of multiple cycles of high vacuum treatment on spore survival, *B. subtilis* spores of different strains were exposed to high vacuum (~2.3 × 10^−6^ Pa) for 1 day in each cycle, followed by return to atmospheric pressure (in ~10 s) for ~30 min before the next cycle of high vacuum treatment. In one experiment spores were suspended in water after each high vacuum treatment, and then redried prior to the next high vacuum treatment. Samples were air-dried spore monolayers (1 × 10^8^ spores) immobilized on stainless steel discs.^29^ To recover spores from the discs, the spore monolayers were covered by a 10% aqueous polyvinyl alcohol solution (PVA) and after drying the spore-PVA layer was removed as described,^2^ and was suspended in 1 ml of sterile distilled water, resulting in >95% recovery of the spores. This procedure has no geno- or cytotoxic effect on spore viability.^43^ The CFU before and after each treatment were determined. In brief, the viability of control and high vacuum-treated spores suspended in water as described above was determined by serial dilution of samples in water, and application of aliquots to LB medium plates which were incubated at 37 °C for 24 to 36 h and then colonies were counted.^20,35,36^ Spores’ CaDPA content was measured using LTRS as previously described.^40,41^ Individual spores suspended in water were randomly trapped by a 780-nm laser beam, and Raman scattering excited by the same laser beam was acquired by a CCD. CaDPA levels in individual trapped spores were determined from the intensity of the CaDPA-specific Raman band at 1,017 cm^−1^ relative to that of CaDPA standards.

### Time-lapse differential interference contrast (DIC) microscopy monitoring of individual spore’s germination

Spore germination was carried out with different types and concentrations of nutrient and non-nutrient germinants, including: (i) 1 mM L-valine in 25 mM K-HEPES buffer (pH 7.4) at 37 °C; (ii) 10 mM AGFK (10 mM each of L-asparagine, D-glucose, D-fructose, and K^+^) in 25 mM K-HEPES buffer (pH 7.4) at 37 °C; (iii) 50 mM CaDPA (pH 7.5) at room temperature; or (iv) 1.0 mM dodecylamine in 25 mM K-HEPES buffer (pH 7.4) at 50 °C. Heat activation of spores in water was carried out at 70 °C for 30 min followed by placing on ice for >15 min prior to L-valine- and AGFK-germination experiments, but this is not needed for CaDPA and dodecylamine germination.^23^

Analysis of the germination of multiple individual spores by DIC microscopy was as described previously.^44,45^ In brief, spores (~1 µl of ~10^7^–10^8^ spores/ml in water) were spread on the surface of a microscope coverslip that was then dried in a vacuum desiccator for 5 to 10 min. The coverslip was then mounted on and sealed to a microscope sample holder kept at a constant temperature. After adding preheated germinant solution to spores on the coverslips, a digital CCD camera (12 bits, 1392 by 1040 pixels) was used to record DIC images at a rate of 1 frame every 15 s for 120 to 180 min. The analysis of DIC images was performed using a program written in Matlab to locate each spore’s position and to calculate the averaged pixel intensity of an area with a 20 pixel diameter that covered the whole individual spore on a DIC image. The DIC image intensity of each individual spore was plotted as a function of the incubation time with a resolution of 15 s. The initial intensity at *T*_0_ was normalized to 1, and the intensity at the end of measurements was normalized to zero. From the time-lapse DIC image intensity, we can determine the time of completion of the rapid fall of ~75% in a spore’s DIC image intensity, consistent with the period of release of almost all of a spore’s CaDPA; the end of this period is defined as *T*_release_, as confirmed by LTRS.^44^ CaDPA release and spore cortex PG hydrolysis kinetics during the germination of individual spores are described by a number of parameters as described previously.^23,24^

### Live-cell imaging of germination, outgrowth, and elongation and growth of single spores incubated on LB agar

For analysis of the germination, outgrowth, elongation, and subsequent vegetative growth of untreated or high vacuum-treated spores, aliquots of spores in water were applied to microscope slides, which were dried under low vacuum, and slides were mounted on a microscope sample holder kept at 37 °C, all as described above. Approximately 250 μL of melted Luria-Bertani (LB)^46^ medium agar at ~60 °C was added on the top of the spores on the coverslip to form an agar pad with a thickness of ~3 mm. Some agar on the side of the pad was removed to form a small hole which contained air and then a second coverslip was applied to seal the top of the agar pad thus preparing the spores for DIC or bright-field microscopy, as described above. A digital CCD camera (12 bits, 1392 by 1040 pixels) was used to record the bright-field images of the spores on the coverslip at a rate of 1 frame every 15 s or 60 s for 4 to 15 h. A home-made auto-focusing system was developed to lock-in the live-cell imaging in focus for more than 15 h.^44^ These images were analyzed with a program written in Matlab as described above. In these measurements, the fates of ≥1000 individual spores were monitored.

In order to measure the stress resistance of high vacuum-treated spores, untreated and high vacuum-treated spores were exposed to dry heat at 140 °C for 7 min and then spore germination with 1 mM L-valine and subsequent growth were analyzed on the LB medium agar pad as described above. The germination and subsequent growth of untreated and high vacuum-treated spores were also examined and analyzed on an LB medium agar pad with 1 M NaCl as described above.

### Statistical analyses

For all germination experiments, ≥300 spores were examined, and because of intrinsic heterogeneity of spore germination, significance analysis were not suitable for germination parameter values. For spore viability analyses on agar plates, three replicates were performed and statistical significance was analyzed using Student’s *t* test. With regards to spore germination, outgrowth, elongation, and vegetative growth, 5 images (≥1000 spores) were examined.

## Electronic supplementary material

Supplementary material

Supplementary Video 1

Supplementary Video 2

## Data Availability

All relevant data are available from the corresponding author.

## Electronic supplementary material

**Supplementary information** accompanies the paper on the *npj Microgravity* website (10.1038/s41526-018-0059-7).
