# Supplementary material for: Single-cell analysis reveals individual spore responses to simulated space vacuum
Source: NPJ Microgravity. 2018 Dec 4;4:26. doi: 10.1038/s41526-018-0059-7 (PMC6279783; doi:10.1038/s41526-018-0059-7)
Supplement: Supplementary file 1 — Supplementary material [file 41526_2018_59_MOESM1_ESM.docx]

**Supplementary material**

**Single-cell analysis reveals individual spore responses to simulated space vacuum**

Lin He^1,2†^, Shiwei Wang^3†^, Marta Cortesão^4^, Muying Wu^1^, Ralf Moeller^4^, Peter Setlow^5^, and Yong-qing Li^1,2^^[[1]](#footnote-1)^*

^1^School of Electronic Engineering, Dongguan University of Technology, Dongguan, Guangdong, P.R. China;

^2^Department of Physics, East Carolina University, Greenville, North Carolina 27858-4353, USA; ^3^School of Chemical Engineering and Energy Technology, Dongguan University of Technology, Dongguan, P.R. China;

^4^German Aerospace Center (DLR e.V.), Institute of Aerospace Medicine, Radiation Biology, Department, Space Microbiology Research Group, Cologne (Koeln), Germany;

^5^Department of Molecular Biology and Biophysics, UConn Health, Farmington, Connecticut 06030-3305, USA

**Supplementary Figure 1 Experimental system for the measurement of Raman spectra of individual spores in a high vacuum environment.** The spores on a quartz coverslip were placed inside a vacuum chamber pumped with a Turbo pumping station, and the spores in the high vacuum environment were examined by micro-Raman spectroscopy, all as described in Methods.


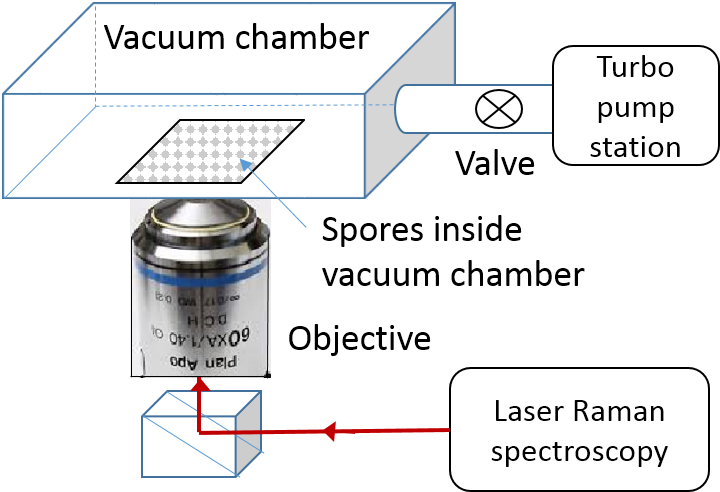


**Supplementary Figure 2 L-Valine germination of multiple individual *Bacillus subtilis* PS533 spores with or without high vacuum treatment.** Germination of ~500 spores was monitored, and image intensities of 10-12 randomly chosen individual spores, either untreated spores (A), 1 day high vacuum-treated spores (B), or 10 day high vacuum-treated spores (C) were obtained as described in Methods. Times of T_1_, T_lag_, T_release_ and T_lys_ are denoted by arrows for one germinating spore in each panel.









**Supplementary Figure 3 AGFK germination of multiple individual *Bacillus subtilis* PS533 spores with or without high vacuum treatment.** Germination of ~ 500 spores was monitored, and image intensities of 10-12 randomly chosen individual spores, either untreated spores (A), 1 day high vacuum-treated spores (B), or 10 day high vacuum-treated spores (C) were obtained as described in Methods. Times of T_1_, T_lag_, T_release_ and T_lys_ are denoted by arrows for one germinating spore in each panel.









**Supplementary Figure 4 CaDPA germination of multiple individual *Bacillus subtilis* PS533 spores with or without high vacuum treatment.** Germination of ~ 500 spores was monitored, and image intensities of 10-12 randomly chosen individual spores, either untreated spores (A), 1 day high vacuum-treated spores (B), or 10 day high vacuum-treated spores (C) were obtained as described in Methods. Times of T_1_, T_lag_, T_release_ and T_lys_ are denoted by arrows for one germinating spore in each panel.









**Supplementary Figure 5 Dodecylamine germination of multiple individual *Bacillus subtilis* PS533 spores with or without high vacuum treatment.** Germination of ~ 500 spores was monitored, and image intensities of 10-12 randomly chosen individual spores, either untreated spores (A), 1 day high vacuum-treated spores (B), or 10 day high vacuum-treated spores (C) were obtained as described in Methods. Times of T_1_, T_lag_, T_release_ and T_lys_ are denoted by arrows for one germinating spore in each panel.









**Supplementary Table 1 Spore survival of *B. subtilis* PS533 spores (wild-type) after multiple cycles of high vacuum treatment with full hydration between cycles^1^.**

| Vacuum treatments | Spore surviving fraction (%) |
| --- | --- |
| 1^st^ cycle | 68.5 ± 15.6 |
| 2^nd^ cycle | 21.3 ± 9.0 |
| 5^th^ cycle | 19.1 ± 7.2 |

^1^Samples of 100 μl of *B. subtilis* PS533 spores (~10^8^ spores/mL) were centrifuged at 10,000 rpm for 5 min, the supernatant fluid removed, and the pellet dried in a vacuum desicator. The dried spores were then exposed to high vacuum (~10^-5^ Pa) or to ambient laboratory conditions (control) for 6 h, followed by return to atmospheric pressure and fully hydration in 100 μl of distill water for > 6 h before the next cycle of high vacuum treatment. Control spore samples of different cycles were treated by the same procedures, but without exposure to high vacuum. CFU before and after each treatment were determined relative to CFU for control samples, which lost no viability. All experiments were conducted in triplicate, and standard deviations for all data are shown.

Supplementary Movie 1. This movie shows the germination, outgrowth, and vegetative growth of individual untreated *B. subtilis* PS533 (wild-type) spores at 37°C on a LB medium agar pad as described in Methods. Time-lapse bright-field images were recorded at a rate of 1 frame every 15 s or 60 s for 5 h. These images were analyzed with a program written in Matlab as described in Methods.

Supplementary Movie 2. This movie shows the germination, outgrowth, and vegetative growth of individual *B. subtilis* PS533 (wild-type) spores that were treated in high vacuum for 10 days and then treated at 140°C for 7 min, at 37°C on a LB medium agar pad as described in Methods. Time-lapse live-cell images were recorded at a rate of 1 frame every 60 s for 10 h. These images were analyzed with a program written in Matlab as described in Methods. While most treated spores germinated, only few spores proceeded into vegetative growth.

1. ^†^Equal contribution.

   *Address correspondence to Yong-qing Li, East Carolina University, Department of Physics, Greenville, North Carolina 27858-4353 United States of America; Tel: 252-328-1858; Fax: 252-328-6314; E-mail: liy@ecu.edu [↑](#footnote-ref-1)
